# Assessment of Functional Capacity of Immune System in Patients with Multiple Sclerosis using QuantiFERON Monitor

**DOI:** 10.1155/2023/4653627

**Published:** 2023-04-07

**Authors:** Zbysek Pavelek, Ondrej Soucek, Jan Krejsek, Ilona Sejkorova, Oldrich Vysata, Blanka Klimová, Francesco Angelucci, Pavel Stourac, Martin Valis, Marek Peterka, Lukáš Sobisek, Michal Novotny

**Affiliations:** ^1^Department of Neurology, Faculty of Medicine and University Hospital Hradec Králové, Charles University in Prague, Hradec Králové, Czech Republic; ^2^Department of Clinical Immunology and Allergology, University Hospital Hradec Králové, Hradec Králové, Czech Republic; ^3^Department of Neurology, University Hospital and Masaryk University, Brno, Czech Republic; ^4^Department of Neurology, Faculty of Medicine and University Hospital Plzen, Charles University in Prague, Plzeň, Czech Republic

## Abstract

**Background:**

The QuantiFERON®-Monitor (QFM) is an assay that measures interferon-*γ* production and was developed to provide an objective marker of complex immune response. In this study, we evaluated the use of the QFM test in patients with two forms of multiple sclerosis (MS), relapsing–remitting form treated with fingolimod (fMS) and secondarily progressive form not treated pharmacologically (pMS), and in healthy controls (HC). We hypothesized that IFN-*γ* levels would be lower in those subjects who are relatively more immunosuppressed and higher in those with normal or activated immune function.

**Methods:**

This single-center observational study was conducted from November 2020 to October 2021 and compared results in three groups of patients: 86 healthy controls, 96 patients with pMS, and 78 fMS. Combination of lyophilized stimulants was added to 1 ml heparinized whole blood within 8 hr of collection. Plasmatic IFN-*γ* was measured using the ELISA kit for the QFM and data were obtained in IU/ml.

**Results:**

The results showed that controls had nearly 2-fold higher levels of IFN-*γ* (QFM score) in median (q25, q75) 228.00 (112.20, 358.67) than the MS patient groups: pMS 144.80 (31.23, 302.00); fMS 130.50 (39.95, 217.07) which is statistically significant difference *P*-value: HC vs. pMS = 0.0071; HC vs. fMS = 0.0468. This result was also confirmed by a validation analysis to exclude impact of variable factors, such as disease duration and Expanded Disability Status Scale scores.

**Conclusions:**

Results showed that controls had higher levels of IFN-*γ* production than the MS patient groups and suggest that MS patients included in this study have a lower ability of immune system activation than HC. Results confirm that fingolimod is able to suppress production of IFN-*γ*. The fact that the QFM score of MS patients is significantly lower than that of HC may indicate a dysfunctional state of the immune system in baseline conditions.

## 1. Introduction

Multiple sclerosis (MS) is an immunopathological and neurodegenerative disease that affects the central nervous system (CNS) [1]. It is characterized by an abnormal activity of the immune system that attacks some CNS components mistaking them for foreign agents. The resulting inflammatory process can damage both the myelin sheath that surrounds and insulates the nerve fibers and the cells specialized in its production, oligodendrocytes, and eventually, it damages the nerve fibers [2]. This process, called demyelination, can result in myelin loss or injury in the white matter, which are referred to as plaques [2].

The natural course of multiple sclerosis is unpredictable. Some people can feel well for many years, while others can quickly develop disabilities. The disease can develop in two forms: either with acute attacks followed by remission or with a gradual progression [3]. In the first case, it is called relapsing–remitting MS (RRMS). Many RRMS patients, but not all, develop the so-called secondarily progressive form (pMS) of MS characterized by a lower frequency (even disappearance) of acute attacks and a continuous functional worsening in the course of RRMS, usually after 15–20 years.

In the studies of MS, it is of paramount importance to evaluate the immunological status of the patient, to verify the disease progression, and to assess the therapy effectiveness [4]. As there is strong evidence that IFN-gamma (IFN-*γ*) is the main cytokine promoting MS disease, our work was focused on it. IFN-*γ* is a cytokine that is produced by activated T lymphocytes and NK cells. IFN-*γ* has numerous immunomodulating and proinflammatory effects, and its level is an important prognostic factor in MS patients. It has been reported that its systemic administration worsen the original MS disease; its increased production has been described before MS exacerbations; it has been found in lesions in patients with MS; it is thought to induce oligodendrocyte death by apoptosis; MS patients have been shown to have an increased number of mononuclear IFN-*γ* secreting cells, as well as a significantly increased level of IFN-*γ* mRNA-expressing mononuclear cells (both in blood and cerebrospinal fluid) [5].

The QuantiFERON®-Monitor (QFM, Qiagen, Germantown, MD, USA) is a novel whole blood immune function assay that measures IFN-*γ* production following the stimulation of innate cells with R848 compound and adaptive immune T cells using antiCD3 monoclonal antibody [6]. The dual stimulation with innate and adaptive ligands confers a significant advantage over single-stimulant assays. The test is performed using the heparinized whole blood with minimal laboratory processing and measurement of INF-*γ* as it is based on the same platform as the widely available QMF Gold assay kit [7].

Although QFM was developed to provide an objective marker of the complex immune response [6, 8] and has so far only been used in transplantation (postallogeneic hematopoietic cell transplantation [9], lung transplantation [6], kidney transplantation [10]), infectious medicine (HIV [11], COVID-19 [12]), and hepatology (cirrhosis [13]), for assessment in neurology (MS patients) has never been used before.

In this study, we were the first researchers to evaluate the use of the QFM test in patients with two forms of MS, fMS [14] and pMS not treated pharmacologically, and in healthy controls (HC). We hypothesized that IFN-*γ* levels would be lower in those subjects who are relatively more immunosuppressed and higher in those with normal or activated immune function.

## 2. Materials and Methods

### 2.1. Study Population

This single-center, observational study using the QFM was conducted from November 2020 to October 2021. In total, 260 participants were enrolled in the study. The study group comprised 86 HC, 96 patients with pMS, and 78 RRMS patients treated with fingolimod (fMS).

The inclusion criterion for HC was the age in range between 18 and 75 years.

The inclusion criteria for patients with pMS are as follows:

1. The participant had to be between 18 and 75 years of age inclusive at the time of signing the informed consent.
2. The participant had to be previously diagnosed with RRMS in accordance with the 2017 revised McDonald criteria [15].
3. The participant had to be currently diagnosed with pMS in accordance with the clinical course criteria [16], revised in 2013 [17].
4. Absence of the clinical relapses for at least 24 months.
5. No administration of any of the following drugs: intravenous immunoglobulin, dimethyl fumarate, fingolimod, teriflunomide, azathioprine, mycophenolate mofetil, methotrexate, and B-cell depleting therapies, such as ocrelizumab and rituximab 12 months prior the entry to the study; mitoxantrone, cyclophosphamide, cladribine, cyclosporine, and alemtuzumab 2 years prior the entry to the study; treatment with methylprednisolone, glatiramer acetate, and interferon *β* 3 months prior the entry to the study.

The inclusion criteria for fMS patients are as follows:

1. The participant had to be between 18 and 75 years of age inclusive at the time of signing the informed consent
2. The participant had to be diagnosed with RRMS in accordance with the 2017 revised McDonald criteria [15]
3. Absence of the clinical relapses for at least 12 months
4. Treatment with fingolimod for at least 3 years

### 2.2. Method Procedure

The QFM assay uses a combination of lyophilized stimulants (QFM Monitor LyoSpheres™), which are added to 1 ml heparinized whole blood within 8 hr of collection. The stimulated blood samples were incubated for 16–24 hr at 37°C and then centrifuged. The plasma was removed and used for measurement amount of IFN-*γ* by an enzyme-linked immunosorbent assay technique (ELISA) using the ELISA kit for the QFM (Qiagen, Hilden, Germany) according to the manufacturer's instructions. Data were obtained in IU/ml. The plasma samples were diluted 1 : 10, 1 : 100, 1 : 200, or 1 : 400 and the absorbance values were read at 450 nm using a Microplate Reader MRX (Dynex Technologies, Inc., Chantilly, VA, USA).

### 2.3. Objective

The objective of this study is to describe and compare QFM testing sampling results between studied populations. The respective endpoint is measured QFM score.

### 2.4. Statistical Analysis

All characteristics are summarized for the whole sample (260) and for each group individually as mean (standard deviation) and median (p25 = lower quartile, p75 = upper quartile) (median), except for categorical variables gender and previous treatment, which are summarized by frequency and percentage. The distribution of the studied endpoint (QFM) is described more in detail by cumulative proportions and shown by violin plots. The statistical significance was assessed by *P*-value only for studied endpoint QFM score.

For other characteristics/covariates, *P*-value presented as descriptive statistics to assess effect size (standardized difference). The standardized groups' differences, i.e., effect size was assessed as *P*-value. *P*-value was computed for gender by performing *χ*^2^ test for independence in contingency table. The effect size (*P*-value) for the previous treatment was not performed due to the lack of observations. The normality of continuous variables was graphically investigated and formally tested by Liliefors test for normality. The effect size between MS patient groups in Expanded Disability Status Scale (EDSS) was assessed by parametric *t*-test. The effect size for age and statistical significance for QuantiFERON test were assessed by using parametric ANOVA. The positively skewed values of QuantiFERON were normalized by logarithm before statistical testing. A post hoc pairwise comparison of the QFM results was run by parametric testing using *t*-test Tukey correction for the false discovery rate.

The validation analysis was conducted to adjust the groups' effect on QFM scores for covariates: age, gender, MS duration, and EDSS. In order to perform the validation, a multiple linear regression model (OLS) for logarithmized test results on group, gender, and age was made for all three groups; and OLS adjusted for gender, age, MS duration, and EDSS for MS patients' groups. In order to reduce the risk of covariates bias was preferred adjusted regression model to the propensity score matching method due to the risk of vast drop out leading in obtaining very small samples for comparison.

All statistical tests were run with two-tailed alternative hypothesis and with type statistical significance (type I error) at 5%. All *P*-values are corrected for multiple testing by Benjamini–Hochberg procedure. All statistical computations were performed in system R (https://www.r-project.org, version 3.5.2 (2018-12-20)) [18].

## 3. Results

Proportions of females and age distribution vary among individual groups, with percentage of females—87.2% HC; 76.0 pMS; 61.5 fMS (*P*-value = 0.0007); mean (median) age at time of measurement—50.2 (49.0) HC; 49.3 (48.0); 41.9 (43.0) (*P*-value < 0.0001). The group of fMS patients is proportionally less represented by females and on average, it comprises a younger age group. The mean duration of treatment with fingolimod is 4.7 years in this group of MS patients.

The pMS patients have a higher rate of clinical disability (as measured by the EDSS scale) with a median EDSS value of 6.5 and a longer duration of illness with a mean duration of 18.3 years compared to fMS patients, with an EDSS of 4.0 (*P*-value < 0.0001) and a mean duration of illness of 11.5 years (*P*-value < 0.0001). These are statistically significant differences (*P*-value < 0.0001 for EDSS and length of illness). A summary of all observed characteristics and for each group is given in Table 1.

**Figure 1:**
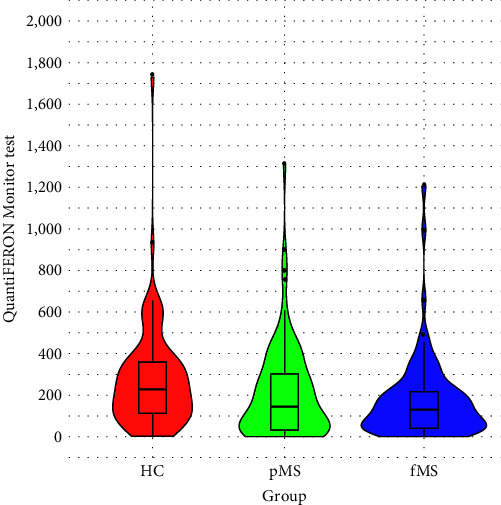
Distribution of measured values of the QFM for each group. Note: the violin plot shows the distribution (probability density) of the QFM scores. Inside the plot, there is a box plot showing the upper and lower quartiles (upper and lower sides of the rectangle) and the median value (bold line inside the diagram). HC, healthy controls; pMS, progressive MS patients; fMS, MS patients treated with fingolimod.

**Figure 2:**
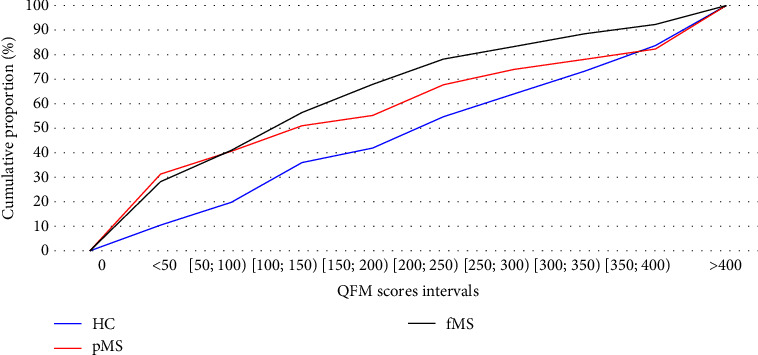
The cumulative distribution function of the QFM scores for each group.

The results of the QFM were different between the MS groups and HC. For HC, the mean (and median) test result is highest at 271.3 (228.0), respectively. In pMS patients, the measured values of 208.6 (144.8) are higher than those of fMS patients 177.1 (130.2). The distribution of QFM result values in each group is shown in Figures 1 and 2 and Table 2. The findings show that HC are more likely to have higher values than MS patients. The interquartile range for HC is between 112 and 359, i.e., 25% of HC had a value <112% and 25% of HC had a value >359. The interquartile range for pMS is 31–302 and for fMS is 40–217.

The difference between HC and pMS and between HC and fMS is statistically significant (*P*-value = 0.0071 and 0.0468, respectively; see Table 3). The cumulative distribution function for HC differs from MS groups, as can be seen in Figure 2. The difference between MS groups is not statistically significant in our patients (*P*-value = 0.8577) in mean values. The results of the pairwise comparison for the QFM are shown in Table 3. The cumulative distribution functions are nearly identical for 50% of patients with lower values (median 144.8 and 130.5 for pMS and fMS). Nevertheless, the sample distribution of values higher than median varies between MS groups (upper quartile = 302.0 for pMS; 217.07 for fMS).

Due to the discrepancies in age, gender, MS duration, and EDSS among the groups, the validation analysis was performed to test the statistical significance of differences in the QFM results after adjusting for these potentially influential factors. The results of the validation regression model with HC as the reference group confirmed a statistically significant difference between HC and pMS (*P*-value = 0.0033) and between HC and fMS (0.0440). The estimation of the regression model is given in Table 4. The difference between MS groups adjusted for age, sex, MS duration, and EDSS is confirmed to be not statistically significant (*P*-value = 0.4501; Table 5).

## 4. Discussion

The purpose of this study was to evaluate the use of the QFM to determine the immune system status of SM patients as compared to HC. Never before has such a study been conducted. QFM been used in transplantation medicine [6, 9, 10]. In their publications, the authors state that QFM can identify excessively immunosuppressed patients, can demonstrate the recovery of patient immunity, or can predict the risk of developing an infection in immunosuppressed patients. At the same time, this tool can be used to adjust the dosage of immunosuppressants or timely addition of antimicrobial therapy. QFM has also found use in infectious medicine [12], where the authors report that the low IFN-*γ* production found after the first week of hospitalization in COVID-19 patients was associated with higher mortality in a subgroup of unvaccinated patients. The authors explain this by a possible higher degree of immune suppression for this subgroup. The level of IFN-*γ* determined by QFM is also lower in cirrhotic patients [13]. This test thus allows an objective determination of an individual's level of immune dysfunction, with the authors stating that a low level of INF was associated with an increased susceptibility to infection.

Our results showed that controls had higher levels of IFN-*γ* production (QFM score) than the MS patient groups. This result was also confirmed by the validation analysis to exclude potentially influential factors, such as disease duration and EDSS scores. On the contrary, there were no significant differences in the QFM scores between pMS and fMS. In spite of nonstatistically significant difference in observed central values (means/medians), our data signalize that QFM scores higher than median may be less variable and closer to median for RRMS patients with fingolimod.

These data suggest that the QFM may be useful to evaluate the immunologic status of SM patients. The test has been successfully used in the assessment of post-transplant immunodepression status [8, 13]. Our data suggest that MS patients included in this study have a lower ability of immune system activation than HC. Addressing the RRMS patients, these results confirm that fingolimod is able to suppress production of IFN-*γ* [19]. This finding is highlighted in recent studies using both rodent cell [20] and human cells models [21]. These data are in line with numerous studies demonstrating the efficacy of this drug evidenced as a statistically significant reduction in the number of active lesions detected by magnetic resonance imaging and recurrences [22]. Two major mega-trials, TRANSFORMS and FREEDOMS [23], have demonstrated a significant reduction in the number of relapses in ∼50% RRMS patients and a reduction in disability progression in at least 30% patients.

Fingolimod (FTY720) is the first of the sphingosine 1-phosphate receptor (S1PR) modulator approved for the treatment of MS patients [24]. It belongs to a novel class of drugs with a new mechanism of action to reduce the immune system attack on the CNS by “sequestering” lymphocytes in the lymph nodes, which is the result of therapeutical manipulation with oriented trafficking of lymphocytes mediated by sphingosine phosphate gradient [25]. Its action prevents lymphocytes to reach the CNS and, thus, preventing attacking the myelin sheaths that protect the nerve fibers, thereby reducing the inflammatory damage. Based on these observations, it is reasonable to think that the reduction in the QFM score in these patients is due to the use of this drug. The reduction in IFN-*γ* certainly reflects the relief of immunopathological activity in these patients.

In MS, the levels of IFN-*γ* are correlated with the frequency of active CNS lesions [26]. IFN-*γ* production by patient-derived T cells is increased before exacerbation (flare) of disease activity [27]. Furthermore, IFN-*γ* can also directly kill oligodendrocytes [28] causing the loss of neuronal myelination observed in the SM patients [29].

Our data confirm that there are no differences between pMS and RRMS patients in the QFM score. Thus, it appears that the QFM cannot distinguish the different forms of MS. We are unable to determine whether the degree of activation or repression of the immune system between these groups of MS patients is different. Patients from both groups were in a nonactive disease state and it would be interesting to verify if the same patient groups in active phase will have different QFM scores. In fact, it is known that IFN-*γ* production is increased in the acute phase of MS but seems to be normal when compared RRMS and pMS patients in remission phase [30]. Since the QFM test is based on IFN-*γ* production after in vitro immune stimulation, it is logical to expect similar values in patients in remission. The fact that the QFM score of these patients is significantly lower than that of HC may instead indicate a dysfunctional state of the immune system in baseline conditions.

## 5. Conclusion

In conclusion, this study demonstrates that the QFM test results vary between HC and MS patients. The test seems to be useful in MS patients in whom it provides a baseline information about the function of immune system. Many confounding factors may influence the QFM score, including immunosuppressive drugs, such as fingolimod. Further studies with different MS groups comparisons are needed to better determine the use of this test as prognostic/predictive biomarker for evaluating the course of MS and/or monitoring the efficacy of new treatments.

## Figures and Tables

**Table 1:** Groups' characteristics and their comparison.

Characteristics	Overall (*N* = 260)			Healthy controls (86)			Progressive MS (96)			MS treated with fingolimod (78)			Effect size (Simultaneous *P*-value)
	*N* (%)	Mean (SD)	Median (p25, p75)	*N* (%)	Mean (SD)	Median (p25, p75)	*N* (%)	Mean (SD)	Median (p25, p75)	*N* (%)	Mean (SD)	Median (p25, p75)	
Gender—number of females (%)	196 (75.4)			75 (87.2)			73 (76.0)			48 (61.5)			0.0007
Age	260 (100.0)	50.15 (11.25)	49 (43, 58)	86 (100.0)	49.31 (9.71)	48.00 (44.00, 55.00)	96 (100.0)	57.57 (8.95)	58.50 (51.75, 64.25)	78 (100.0)	41.94 (9.20)	43.00 (38.00, 47.00)	<0.0001
EDSS	174 (66.9)	5.06 (1.72)	5.5 (4, 6.5)	NA	NA	NA	96 (100.0)	6.09 (1.01)	6.50 (5.50, 6.50)	78 (100.0)	3.79 (1.56)	4.00 (2.12, 5.00)	<0.0001
Disease duration	174 (66.9)	15.24 (7.2)	14 (10.25, 20)	NA	NA	NA	96 (100.0)	18.30 (6.69)	17.00 (12.00, 23.00)	78 (100.0)	11.47 (5.94)	11.00 (6.00, 15.00)	<0.0001
Fingolimod treatment duration (in years)	78 (30.0)	4.68 (1.46)	5 (3, 6)	NA	NA	NA	NA	NA	NA	78 (100.0)	4.68 (1.46)	5.00 (3.00, 6.00)	NA
QFM (results)	260 (100.00)	219.89 (226.65)	168.55 (55.55, 313.4)	86 (100.0)	271.32 (244.34)	228.00 (112.20, 358.67)	96 (100.0)	208.61 (223.65)	144.80 (31.23, 302.00)	78 (100.0)	177.05 (200.50)	130.50 (39.95, 217.07)	0.0064
Previous treatment													NA
DMF	1 (0.4)			NA			0 (0.0)			1 (1.3)			
GA	21 (8.1)			NA			0 (0.0)			21 (26.9)			
GA, IFN	2 (0.8)			NA			0 (0.0)			2 (2.6)			
GA, teriflunomid	1 (0.4)			NA			0 (0.0)			1 (1.3)			
IFN	39 (15.0)			NA			1 (1.0)			38 (48.7)			
IFN, DMF	2 (0.8)			NA			0 (0.0)			2 (2.6)			
IFN, GA	3 (1.2)			NA			0 (0.0)			3 (3.8)			
Teriflunomid	1 (0.4)			NA			0 (0.0)			1 (1.3)			

Note: All characteristics are reported as a mean (standard deviation) and median (p25 = lower quartile, p75 = upper quartile) (median), except categorical variables gender and previous treatment, which are summarized by frequency and percentage. The standardized groups' differences, i.e., effect size was assessed as *P*-value. *P*-value was computed for gender by performing *χ*^2^ test for independence in contingency table. The effect size (*P*-value) for previous treatment was not performed due to the lack of observations. The effect size for age and statistical significance for QuantiFERON test were assessed by parametric ANOVA. The positively skewed values of QuantiFERON were normalized by logarithm before statistical testing. The difference between MS patient groups in EDSS was assessed by *P*-value from parametric *t*-test. EDSS, Expanded Disability Status Scale.

**Table 2:** The cumulative distribution function of the QFM scores for each group.

Quantiles/interval values	HC	pMS	fMS
Quantiles			
Min	2.03	0.76	1.06
p25	112.2	31.23	39.95
Median	228	144.8	130.5
p75	358.67	302	217.07
Max	1730.4	1307.6	1206.4
Cumulative distribution: frequencies (%)			
<50	9 (10.5)	30 (31.3)	22 (28.2)
[50; 100)	17 (19.8)	39 (40.6)	32 (41.0)
[100; 150)	31 (36.0)	49 (51.0)	44 (56.4)
[150; 200)	36 (41.9)	53 (55.2)	53 (67.9)
[200; 250)	47 (54.7)	65 (67.7)	61 (78.2)
[250; 300)	55 (64.0)	71 (74.0)	65 (83.3)
[300; 350)	63 (73.3)	75 (78.1)	69 (88.5)
[350; 400)	72 (83.7)	79 (82.3)	72 (92.3)
>400	86 (100)	96 (100)	78 (100)

Note: This table shows cumulative distribution of the QFM scores. HC, healthy controls; pMS, progressive MS patients; fMS, MS patients treated with fingolimod; p25, lower quartile; p75, upper quartile.

**Table 3:** Pairwise comparison of the QuantiFERON Monitor test results.

Groups	diff	lwr	upr	Effect size (*P*-value)
Healthy controls vs. progressive MS	−0.6495209	−1.1511570	−0.147884809	0.0070598
Healthy controls vs. MS treated with fingolimod	−0.5341525	−1.0624314	−0.005873529	0.0468285
Progressive MS vs. MS treated with fingolimod	0.1153684	−0.3996581	0.630394952	0.8576530

Note: This table shows mean differences between groups (diff) and 95% confidence interval estimates (lwr = lower, upr = upper) for average differences. Each *P*-value, which represents the result of a pairwise parametric testing using *t*-test, is reported after Tukey correction for the false discovery rate.

**Table 4:** Multiple regression model for logarithmized QuantiFERON Monitor test on group, gender, and age.

Coefficient	Estimate	Std. error	*t*-value	Effect size (*P*-value)
(Intercept)	4.880289	0.538352	9.065	<0.0001
Group (progressive MS)	−0.680075	0.229022	−2.969	0.0033
Group (MS treated with fingolimod)	−0.490890	0.242530	−2.024	0.0440
Age	0.004305	0.009668	0.445	0.6565
Gender (female)	0.044792	0.213154	0.210	0.8337

Note: This table shows coefficients estimates of linear multiple regression model (ordinary least squares method for estimates) for logarithmized results of the QuantiFERON test. (Intercept) is the estimated coefficient for reference group (healthy controls group, males).

**Table 5:** Multiple regression model to compare logarithmized QuantiFERON Monitor test values in MS patients (pMS, fMS) adjusted for gender, age, MS duration, and EDSS.

Coefficient	Estimate	Std. error	*t*-value	Effect size (*P*-value)
(Intercept)	3.661247	0.867172	4.222	<0.0001
Group (MS treated with fingolimod)	0.262901	0.347292	0.757	0.4501
Age	0.021007	0.013474	1.559	0.1209
Gender (female)	0.260875	0.256779	1.016	0.3111
MS duration (in years)	−0.034204	0.020623	−1.659	0.0991
EDSS	0.006415	0.101250	0.063	0.9496

EDSS, Expanded Disability Status Scale.

## Data Availability

The data that support the findings of this study are available from the corresponding author upon reasonable request.
